# Supplementary material for: Comparative multiomics analysis of cell physiological state after culture in a basket bioreactor
Source: Sci Rep. 2022 Nov 23;12:20161. doi: 10.1038/s41598-022-24687-4 (PMC9686226; doi:10.1038/s41598-022-24687-4)

66048.1:132569242:132569440:-@NW_023666048.1:132566587:132566714:-@NW_023666048.1:132566257:132

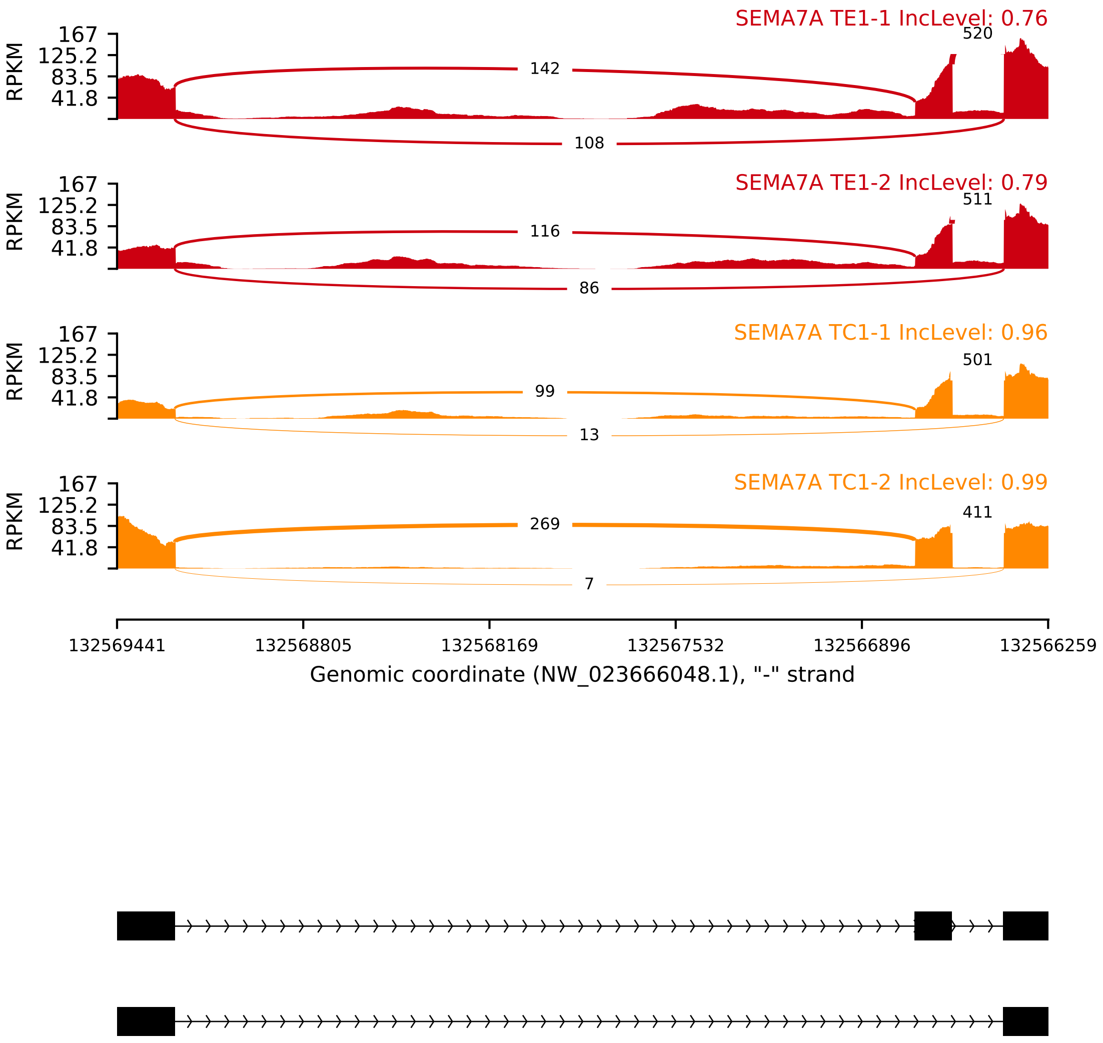

Supplement: Supplementary file 1 — Supplementary Information 1. [file 41598_2022_24687_MOESM1_ESM.zip › raw data/Transcriptomic raw data/7.AS/2.dasplot/TE1vsTC1/SE/8_SEMA7A_NW_023666048.1_132569242_132569440_-@NW_023666048.1_132566587_132566714_-@NW_023666048.1_132566257_132566411_-.pdf]
